# Nisin inducible production of listeriolysin O in *Lactococcus lactis *NZ9000

**DOI:** 10.1186/1475-2859-7-24

**Published:** 2008-07-29

**Authors:** Mohammed Bahey-El-Din, Brendan T Griffin, Cormac GM Gahan

**Affiliations:** 1 Department of Microbiology, University College Cork, Cork, Ireland; 2 School of Pharmacy, University College Cork, Cork, Ireland; 3 Alimentary Pharmabiotic Centre, University College Cork, Cork, Ireland

## Abstract

**Background:**

*Listeria monocytogenes *is a well-characterized food-borne pathogen that infects pregnant women and immunocompromised individuals. Listeriolysin O (LLO) is the major virulence factor of the pathogen and is often used as a diagnostic marker for detection of *L. monocytogenes*. In addition, LLO represents a potent antigen driving T cell-mediated immunity during infection. In the present work, *Lactococcus lactis *NZ9000 was used as an expression host to hyper-produce LLO under inducible conditions using the NICE (NIsin Controlled Expression) system. We created a modified pNZ8048 vector encoding a six-His-tagged LLO downstream of the strong inducible PnisA promoter.

**Results:**

The constructed vector (pNZPnisA:CYTO-LLO) was expressed in *L. lactis *NZ9000 and was best induced at mid-log phase with 0.2% v/v nisin for 4 h statically at 30°C. Purification of the His-tagged LLO was accomplished by Ni-NTA affinity chromatography and functionality was confirmed through haemolytic assays. Total LLO yield (measured as total protein content) was 4.43–5.9 mg per litre culture and the haemolytic activity was still detectable after 8 months of storage at 4°C.

**Conclusion:**

The LLO production method described in this work provides an approach to efficient LLO production in the Gram-positive *Lactococcus *bacterium to yield a significant source of the protein for research and diagnostic applications. Expression of LLO in *L. lactis *has a number of benefits over *E. coli *which may facilitate both *in vivo *and *in vitro *applications of this system.

## Background

*Listeria monocytogenes *is the causative agent of listeriosis, a food-borne disease generally associated with the consumption of contaminated ready-to-eat food products. Listeriosis affects mainly immunocompromised individuals and the outcome of infection includes spontaneous miscarriage in pregnant women, and meningitis in the newborn [1]. *L. monocytogenes *is common in the environment and can be found in the gastrointestinal tract of approximately 5% of healthy persons. Symptomatic infection occurs in individuals with immunosuppression including patients with AIDS and organ transplant recipients [2]. Listeriosis is usually a severe disease with a mean mortality rate in humans of 20 to 30% [3]. Several common-source outbreaks of listeriosis have been linked to consumption of *Listeria*-contaminated foods (frankfurters, pâté, pasteurised milk and soft-cheese) [2]. Controlled good manufacturing procedures and early detection of *Listeria *contamination in food are of great importance in preventing such outbreaks.

The major virulence factor of *L. monocytogenes *is the haemolysin listeriolysin O (LLO). Upon internalisation of the bacterium within the host cell phagolysosome, rapid acidification (~pH 5.5) activates LLO. The protein then interacts with vacuolar membrane cholesterol, oligomerizes and produces pores in the phagolysosomal membrane permitting bacterial escape to the cytoplasm [3]. LLO is therefore critical to *L. monocytogenes *infection as it facilitates intracellular pathogenesis and subsequent spread of the infection to other tissues.

Enhanced expression systems for *in vitro *production of LLO have a number of potential applications. Since LLO is associated solely with the pathogenic strain *L. monocytogenes*, the use of anti-LLO antibodies to identify the pathogen in diagnostic or food samples has demonstrated significant potential [4]. Pure LLO could also find an application in assays to determine prior exposure and immunity to the pathogen in humans or animals [5]. Finally, LLO is recognised as an immunodominant antigen by T cells generated during infection [6]. Modified strains expressing LLO may therefore have applications as vaccines for delivering this *Listeria*-specific antigen to the host immune system.

We have chosen to utilise *Lactococcus lactis *as a vector for hyper-expressing LLO. *L. lactis *is a GRAS (generally regarded as safe) microorganism widely used in the food industry. Recent advances in the molecular characterization of *L. lactis *and the development of *L. lactis*-compatible genetic engineering tools have increased the versatility of this organism as a means of protein production [7-9]. Moreover, the use of lactococci has been extended by others as a live antigen and therapeutic drug delivery system [10,11]. Several protein expression systems, both constitutive and inducible, have been developed in lactococci [8]. However, constitutive systems have a potential disadvantage in that the protein may be more liable to lactococcal degradation, or may prove toxic to the cell if produced in high amounts for extended periods.

One of the best characterized inducible expression systems for use in lactococci is the NICE (NIsin Controlled Expression) system [12,13]. In the present work, we describe high level production of the *L. monocytogenes *LLO protein in *Lactococcus lactis *NZ9000 using the NICE system. The His-tagged LLO was purified by Ni-NTA affinity chromatography to give a considerable yield and high functional haemolytic activity compared to other published methods [14,15]. The significant yield and purity obtained for LLO using the NICE-expression system in *Lactococcus *demonstrate the benefits of this approach for the production of LLO for research and diagnostic purposes. Moreover, the constructed LLO-expressing *L. lactis *strain has the potential to act as a safe live vaccine candidate against listeriosis.

## Methods

### Culture media, antibiotics and incubation conditions

For *L. lactis*, GM17 broth (M17 broth (Oxoid) supplemented with 0.5% glucose) was used as a standard culture medium. Luria-Bertani (LB) broth (10 g peptone from casein, 5 g yeast extract and 5 g sodium chloride per litre) was used for *Escherichia coli *cultures. Solid media were prepared by adding technical agar (Merck) to the corresponding broth with a final concentration of 1.5% w/v. *L. lactis *cultures were incubated statically at 30°C while *E. coli *was incubated at 37°C with shaking when applicable. When required, ampicillin (Amp) was used at a concentration of 100 μg/ml for *E. coli *while chloramphenicol (Cm) was used at 10 μg/ml for both *E. coli *and *L. lactis*.

### Construction of plasmid vector for expression of LLO in *L. lactis *NZ9000

Bacterial strains and vectors used in the present study are described in Table 1 while PCR primers are summarized in Table 2. High fidelity KOD hot start DNA polymerase (Novagen) was used in all PCR reactions throughout the whole procedures following manufacturer's instructions. Restriction enzymes and T4 DNA ligase were purchased from Roche Diagnostics (Mannheim, Germany). The gene encoding listeriolysin O, *hly*, of *L. monocytogenes *EGD-e was PCR-amplified without the signal peptide coding sequence from the chromosomal DNA [GenBank: AL591824] using primers 1 and 2. The resulting PCR product was sequentially digested by *Bam*HI and *Pst*I respectively. Ligation to a similarly digested pQE30 vector (Qiagen) was successfully performed using T4 DNA ligase. The ligation reaction mixture was transformed into chemically-competent *E. coli *BL21 (Novagen) by heat shock following manufacturer's instructions and plated onto LB agar containing 100 μg/ml ampicillin. Positive colonies were identified by PCR and plasmid was extracted from BL21 using Qiagen Miniprep Kit (Qiagen). The resulting plasmid (pQE30/*hly*) had an N-terminus six-histidine tagged *hly *gene and the correct nucleotide sequence was confirmed by DNA sequencing (Lark Technologies Inc., UK). pQE30/*hly *was utilised as a template for further cloning steps outlined below.

**Table 1:** Bacterial strains and plasmid vectors used in the present study

**Strain or plasmid** ** name**	**Description**	**Reference** ** or source**
*E. coli *Top10	Chemically-competent intermediate host, plasmid free	Invitrogen
*E. coli *BL21	Chemically-competent *E. coli*, used in this study as an intermediate host for pQE30, plasmid free	Novagen
*Listeria monocytogenes EGD-e *serovar 1/2a	Wild type *Listeria monocytogenes*	[32]
*Lactococcus lactis *NZ9700	Nisin producer strain	[13]
*Lactococcus lactis *NZ9000	*L. lactis *subsp. *Cremoris *MG1363 carrying nisRK on the chromosome	[13]
*L. lactis *NZ9000 (pNZPnisA:CYTO-LLO)	*Lactococcus lactis *NZ9000 harbouring pNZPnisA:CYTO-LLO plasmid and over-expressing LLO upon nisin induction	This study
pQE30	Expression vector using phage T5 promoter and adding an N-terminus six-His tag to the expressed protein, Amp^R ^(ampicillin resistant)	Qiagen
pQE30/*hly*	pQE30 vector with *hly *gene (without signal sequence) inserted between *Bam*HI and *Pst*I restriction sites.	This study
pNZ8048	*E. coli-L. lactis *shuttle vector containing PnisA promoter and start codon in *Nco*I site, Cm^R ^(chloramphenicol resistant)	[13]
pNZPnisA:CYTO-LLO	Modified pNZ8048 containing PnisA promoter (*Nco*I site eliminated) with downstream His-tagged *hly *gene, Cm^R^	This study

**Table 2:** Oligonucleotide primers used in this study.

**Primer** **code** ** number**	**Primer** ** name**	**Primer sequence (5'-3') (restriction enzyme site or overhang)**^a^
1	pQE30 forward primer	GAA*GGATCC*GATGCATCTGCATTCAATAAAG (*Bam*HI site)
2	pQE30 reverse primer	ACGC*CTGCAG*TTCGATTGGATTATCTACTTTATTA (*Pst*I site)
3	PnisA forward primer	CCA*AGATCT*AGTCTTATAACTATACTG (*Bgl*II site)
4	PnisA reverse primer (*hly *overhang)	GGTGATGTCCCATTTTGAGTGCCTCCTTATAATTTATTTTG (*hly *overhang)
5	*hly *forward primer (PnisA overhang)	AGGCACTCAAAATGGGACATCACCATCACCATCACGGA (PnisA overhang)
6	*hly *reverse primer	AGTC*GGTACC*TTATTCGATTGGATTATCTAC (*Kpn*I site)

(^a^) Restriction enzyme recognition sites are italicized and underlined while SOE overhangs are underlined.

**Figure 1:**
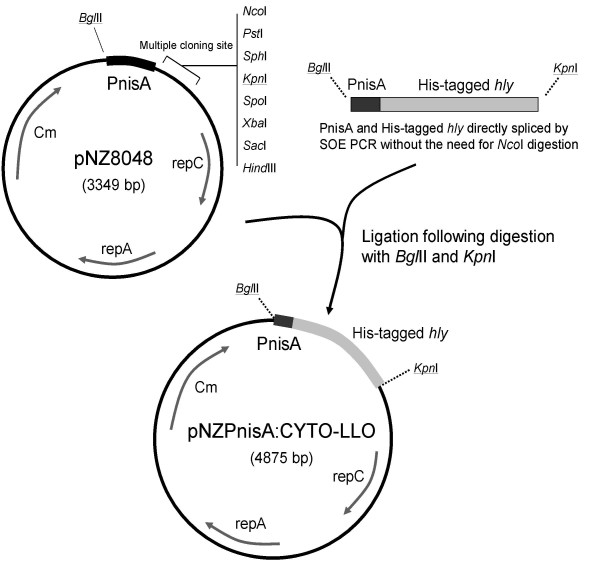
**Schematic representation of the construction of the pNZPnisA:CYTO-LLO vector**.

**Figure 2:**
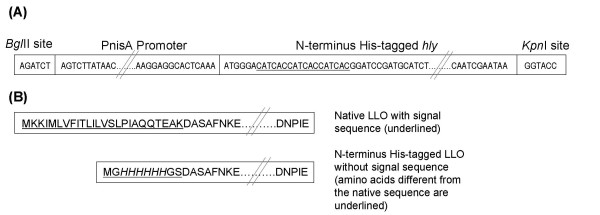
**Illustrative partial DNA and amino acid sequence of the constructed vector and LLO respectively**. (A) Illustrative DNA sequence of the SOE (splicing by overlap extension) PCR product consisting of the PnisA promoter spliced to downstream six-His-tagged *hly *gene of *L. monocytogenes *EGD-e. Flanking restriction sites of *Bgl*II and *Kpn*I exist on the PnisA and *hly *sides respectively. The six-His-tag codons are underlined. (B) Comparison between the amino acid sequence of native LLO (*L. monocytogenes *EGD-e) with the signal sequence and the constructed six-His-tagged LLO produced in this study.

The lactococcal plasmid pNZ8048 [13] was manipulated to allow insertion of the His-tagged *hly *gene. Plasmid pNZ8048 contains the PnisA promoter (nisin inducible) with a downstream start codon (ATG) inside an *Nco*I restriction site (CCATGG). However, since the *hly *gene contains an internal *Nco*I restriction site, we modified pNZ8048 to eliminate this NcoI site and added a new start codon in frame with *hly *gene. The Splicing by Overlap Extension (SOE) technique [16] was used to construct an insert containing the His-tagged *hly *gene spliced directly to PnisA promoter without the need for *Nco*I digestion. Two PCR reactions were initially performed: primers 3 and 4 were used to amplify the PnisA promoter from pNZ8048 while primers 5 and 6 were used to amplify *hly *(along with the His-tag) from the constructed pQE30/*hly *plasmid. The hybrid primers 4 and 5 had overhangs (Table 2) which allow the overlap of the previous two PCR products upon combination for the third splicing PCR reaction. This third PCR reaction was done by combining the initial two PCR products in a molar ratio of 1:1 and again PCR-amplified using primers 3 and 6. The resulting PCR spliced product (PnisA with downstream His-tagged *hly*) was sequentially digested by *Bgl*II and *Kpn*I respectively. pNZ8048 was similarly digested with those two restriction enzymes, to remove the PnisA promoter along with the *Nco*I site, and the digested plasmid was agarose gel purified using the Qiagen gel extraction kit (Qiagen). The spliced PCR product was ligated into the digested pNZ8048 using T4 DNA ligase. The ligation reaction was transformed into chemically-competent *E. coli *TOP10 (Invitrogen) following the manufacturer's instructions and plated onto LB agar containing 10 μg/ml chloramphenicol. After incubation at 37°C for 24–48 h, positive colonies were detected by colony PCR and plasmid (designated pNZPnisA:CYTO-LLO) was extracted and correct DNA sequence was confirmed (Lark Technologies Inc., UK). Plasmid pNZPnisA:CYTO-LLO was transformed to electrocompetent *L. lactis *NZ9000, prepared as previously described [17], using Gene Pulser (Biorad) and plated onto GM17 agar containing 10 μg/ml chloramphenicol. After 24 h incubation at 30°C, colonies were checked by colony PCR and one positive colony was stocked for protein induction and expression. Figures 1 and 2 show an overview of the plasmid vector construction and the sequence of DNA and amino acids of the final His-tagged LLO respectively.

### Protein induction

Filter-sterilized culture supernatant of the nisin-secreting strain *L. lactis *NZ9700 was used as a source of nisin [18]. Sterile culture supernatant of *L. lactis *NZ9700 was stored in small aliquots at -20°C and one aliquot was thawed and used as required. The use of the same filter-sterilized batch of supernatant throughout the study ensured consistency of the nisin content between induction experiments. Overnight culture of *L. lactis *NZ9000 (pNZPnisA:CYTO-LLO) was subcultured into fresh GM17 broth (Cm 10 μg/ml) and incubated statically at 30°C. Nisin was added when the optical density at 600 nm (OD600) reached 0.5. Induction was conducted statically at 30°C after which cells were pelleted (3200 × g for 10 min) and washed once with buffer (50 mM NaH_2_PO_4_, 300 mM NaCl, 10 mM imidazole, pH 8). Pellets were frozen at -80°C prior to sodium dodecylsulphate-polyacrylamide gel electrophoresis (SDS-PAGE) or protein purification.

### SDS-PAGE and Western blot

Frozen induced pellets from 500 ml cultures were thawed on ice and resuspended in 10–15 ml ice-cold lysis buffer (50 mM NaH_2_PO_4_, 300 mM NaCl, 10 mM imidazole, pH 8) containing 30 mg/ml lysozyme (Sigma) and kept for 30 min on ice. This was followed by sonication at maximum amplitude (Soniprep 150, MSE UK Ltd.) using eight ten-second pulses with intervening ten-second pauses on ice. For small volume cultures (10–50 ml), pellets were resuspended in 500 μl lysis buffer in eppendorf tubes and acid-washed glass beads (Sigma) were added followed by beating the tubes in a bead beater (Mini beadbeater-8, Biospec products) for three one-minute beats with one-minute pauses in between on ice. Clear supernatant was obtained after centrifugation at 10000 × g for 30 min at 4°C.

For SDS-PAGE, 12% SDS-polyacrylamide separating gel and 4% stacking gel were used and gels were stained with coomassie blue stain followed by destaining and gel imaging. For Western blot, pre-stained protein marker was used (Amersham Biosciences UK, Ltd) and gels were blotted against a nitrocellulose membrane (Hybond-ECL™, Amersham Biosciences UK, Ltd.) using a semi-dry Western transfer apparatus. Membranes were blocked overnight at 4°C in 5% skimmed milk in TBS buffer (0.8% NaCl, 20 mM Tris-HCl, pH 7.6). Primary rabbit anti-LLO antibody (Diatheva, Italy) and secondary anti-rabbit antibody (Amersham ECL Western Blotting System) were used at 1/1000 and 1/1500 dilutions in 5% and 10% skimmed milk in TBS buffer respectively. Western blot detection was done using Amersham ECL Western Blotting System (Amersham Biosciences UK, Ltd.) using the protocol recommended by the manufacturer.

### Protein purification and quantitation

Clear supernatant obtained after cell lysis (performed as described in the previous section) was passed through a disposable chromatography column (Biorad) containing 2 ml Ni-NTA affinity gel (Qiagen). Column was washed twice with wash buffer (50 mM NaH_2_PO_4_, 300 mM NaCl, 20 mM imidazole, pH 8) then eluted in fractions of 0.5 ml with elution buffer (50 mM NaH_2_PO_4_, 300 mM NaCl, 250 mM imidazole, pH 8). Elutions were checked by SDS-PAGE and combined together and dialysed overnight at 4°C against 2 l of dialysis buffer (500 mM NaCl, 10 mM Na_2_HPO_4_, 0.5 mM EDTA, 0.02% NaN_3_, pH 7.0). Listeriolysin O concentration was measured as total protein concentration by Micro Lowry total protein kit (Sigma) and also by absorbance at 280 nm using the molar extinction coefficient for His-tagged LLO as 71,950 cm^-1 ^(calculated based on the actual sequence of the His-tagged LLO) [19]. The purified protein was stored at 4°C.

### Measurement of haemolytic activity of purified LLO

Haemolytic activity of LLO was measured according to the method described by Khoda et al [20] with slight modification. Briefly, defibrinated sheep blood was centrifuged at 800 × g for 10 min at 4°C, then washed twice with phosphate buffered saline (PBS) (Gibco) pH 5.5. The pelleted red blood cells (RBCs) were diluted with PBS (pH 5.5) to obtain 0.5% RBCs volume/volume (v/v). Aliquots of 100 μl of the RBCs suspension were distributed in 1.5 ml tubes and twofold serial dilutions of LLO (in PBS pH 5.5) was added to each tube to a final volume of 1 ml. LLO final concentrations covered the range from 8 μg ml^-1 ^to 29 pg ml^-1^. A positive control (distilled water, 100% haemolysis) and a negative control (PBS pH 5.5) were also included. Tubes were incubated statically at 37°C for 45 min after which they were centrifuged at 1700 × g for 5 min and supernatants were collected. Absorbance was measured colorimetrically at 415 nm and haemolytic units were calculated. One haemolytic unit (HU) was defined as the amount of protein required to cause 50% haemoglobin release from sheep RBCs as compared to the 100% haemoglobin release of the positive control (distilled water) [20].

## Results

### DNA sequence of the six His-tagged *hly *gene

Native LLO secreted by *Listeria monocytogenes *EGD-e is composed of a secretory signal peptide (25 amino acids) and the actual active portion of LLO (504 amino acids). In the cloning described in this paper we omitted the native secretory signal sequence (coding for the first 25 amino acids) of LLO and added six histidine amino acids (a His-tag) at the N-terminus (Figure 2). DNA sequencing of the constructed pNZPnisA:CYTO-LLO confirmed the addition of the six-His-tag upstream of the *hly *gene and the integrity of the remainder of the *hly *gene sequence. Overall we added a total of 10 amino acids (including the 6 histidine residues) at the N-terminus of LLO, in place of the signal sequence, to facilitate tagging and purification of the protein (Figure 2). These few amino acids did not affect protein activity as shown below.

### Nisin-induced LLO production in *L. lactis *NZ9000 (pNZPnisA:CYTO-LLO)

**Figure 3:**
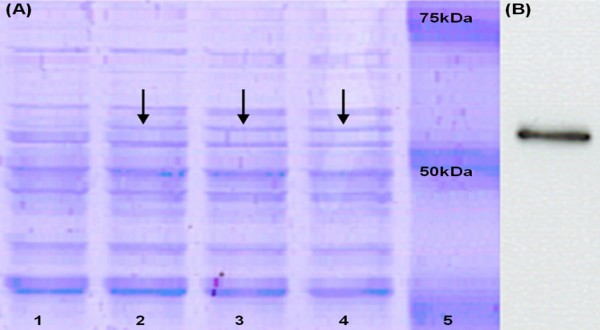
**Optimization of nisin concentration for induction experiments**. (A) SDS-PAGE of the induction experiment for 3 h statically at 30°C without nisin addition (lane 1), and using nisin at concentrations of 0.02% (lane 2), 0.1% (lane 3) and 0.2% (lane 4) (v/v). Lane 5 represents the protein marker. (B) Western blot showing LLO at the expected size of about 57 kDa using rabbit anti-LLO as primary antibody.

We performed a series of induction experiments on *L. lactis *NZ9000 (pNZPnisA:CYTO-LLO) to determine the optimum conditions for protein expression. Preliminary induction time-course experiments showed the optimum duration for nisin-induction to be 3–4 h (data not shown). Consequently, induction was performed at mid-log phase (OD600 = 0.5) for three hours. Initially, different nisin concentrations were examined: 0.02%, 0.1%, and 0.2% (v/v), to determine the optimum concentration for induction and SDS-PAGE was performed to assess the amount of LLO produced (Figure 3A). Subsequently, for protein over-production experiments, 0.2% v/v nisin concentration was chosen for induction to maximize the amount of LLO produced. Western blot was also performed and confirmed the production of pure non-degraded LLO (Figure 3B). It is noteworthy that we detected minimal basal expression of LLO in the uninduced *L. lactis *NZ9000 (pNZPnisA:CYTO-LLO) with no apparent toxicity on bacterial growth (data not shown). The inducer of basal PnisA activity in our experiments in the absence of nisin is not clear although other inducers such as lactose and galactose have been previously reported to induce this promoter at low levels [21]. In our experiments the basal LLO expression was negligible compared to the amount of LLO produced upon nisin induction.

### Purification of LLO and total protein quantitation

**Figure 4:**
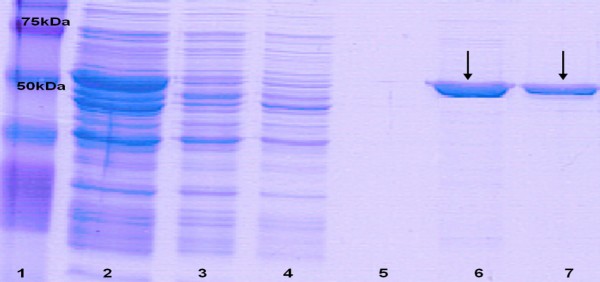
**SDS-PAGE of purified LLO using Ni-NTA affinity chromatography**. Lane 1 is the protein marker, lane 2 is culture lysate of *L. lactis *NZ9000 (pNZPnisA:CYTO-LLO) before passing through the column, lane 3 is the flow-through of the column, lanes 4 and 5 are two successive washes of the columns while lanes 6 and 7 are two successive elutions showing the purified His-tagged LLO.

**Figure 5:**
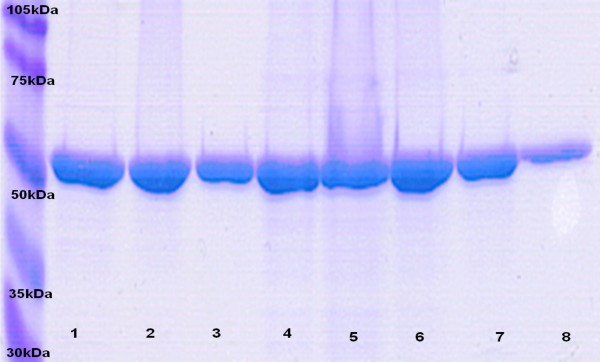
**SDS-PAGE showing pure LLO elutions (500 ml bacterial culture) using the double sonication approach**. Pure LLO was eluted from the Ni-NTA affinity gel after 4 h-0.2% nisin induction at 30°C and double sonication treatment of the induced cells as described in the Methods section. Lanes 1–3: elutions after first sonication treatment. Lanes 4–8: elutions after second sonication treatment.

**Figure 6:**
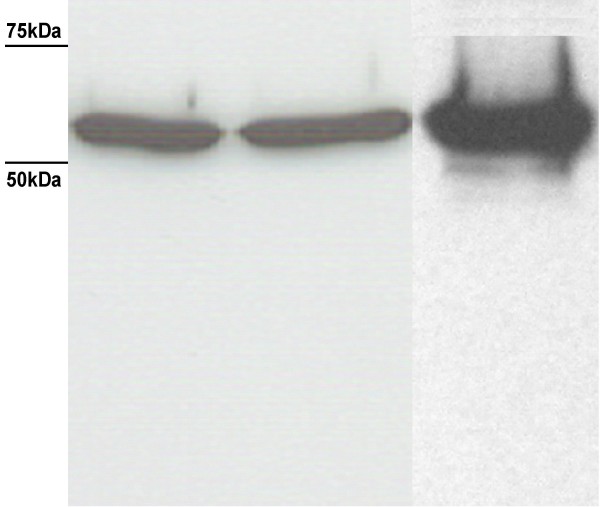
**Western blot showing LLO level and stability after 0.2% nisin induction**. Left lane, LLO after 3 h induction (single sonication); Middle lane, LLO after 4 h induction (single sonication); Right lane, LLO after 4 h induction followed by the double sonication treatment (few degradation bands are observed at slightly lower molecular weights).

To obtain rapid and reproducible purification of LLO, we used the Ni-NTA technology to capture and purify the six-His-tagged protein by affinity chromatography under native purification conditions. Figure 4 shows a representative purification procedure of the His-tagged LLO (about 57 kDa) checked by SDS-PAGE. The average yield of pure LLO (measured as total protein) after 3 h-induction at 30°C using a concentration of 0.2% v/v of nisin was 3 mg per litre culture as measured by both Lowry and A_280 _absorbance methods. When the induction time was increased to 4 h at 30°C, the yield ranged from 4.43–5.9 mg per litre culture. However, to increase the total yield of LLO, we modified the LLO extraction method by applying double sonication to the 4 h-induced pellets of *L. lactis *NZ9000 (pNZPnisA:CYTO-LLO). In brief, after the first sonication was completed (as described in the Methods section), the supernatant was collected for Ni-NTA purification and the remaining pellets were frozen again at -20°C for 24 hours then thawed, resuspended in lysis buffer and sonicated again. Supernatant was collected and then Ni-NTA-gel-purified. This second sonication of the same pellets simply extracted LLO which had not been released from *Lactococcus *cells upon the first treatment. Eluted proteins from the two sonication treatments were tested by SDS-PAGE for purity (Figure 5) then combined and dialysed as mentioned earlier. This double sonication approach increased the total combined yield of LLO to the range of 9.3–12.9 mg per litre culture (average 11.6 ± 1.56 mg per litre culture). However, when the resulting LLO was examined by Western blot, minor lower molecular weight bands appeared below the major LLO band. This indicated slight degradation of LLO upon the double sonication procedure (Figure 6).

To check the functionality of LLO, the haemolytic titre test was carried out and specific LLO haemolytic activity ranged from 5 × 10^5 ^to 5 × 10^7 ^HU per mg total protein. This haemolytic activity was still detectable after at least 8 months of storage at 4°C.

## Discussion

In the present study, we describe the over-expression of the haemolysin listeriolysin O (LLO) of *L. monocytogenes *by the GRAS microorganism *L. lactis *NZ9000. We used a well-defined strong promoter system (the NICE system) in which addition of subinhibitory nisin induces the PnisA promoter for over-expression of downstream genes [12,13]. In the present work, we utilised a nisin concentration of 0.2% v/v for induction which maximized LLO production and was far below the minimum inhibitory concentration (MIC) of nisin against the producing strain (MIC was found to be 3.1% v/v).

The NICE system has been used extensively in the literature to over-produce proteins in lactococci. For instance, *Brucella abortus *GroEL protein was produced in *L. lactis *NZ9000 although it was found that only the secreted rather than the intracellular form was stably produced suggesting a detrimental effect of GroEL protein on *Lactococcus *[22]. In contrast, *Giardia lamblia *cyst wall protein 2 (CWP2) was successfully produced in different cell compartments (intracellular, secreted, and cell-surface anchored) by use of appropriately designed vectors without any detrimental effects on the *Lactococcus *producer. Lactococci expressing CWP2 on their surface could elicit CWP2-specific IgA antibodies and reduced cyst shedding in a murine *Giardia *challenge model [23].

Here, we demonstrate the use of the NICE system to successfully over-express LLO of *L. monocytogenes *in the intracellular compartment of *L. lactis *NZ9000. Several previous studies examined the over-production of LLO in different host cells and expression systems (summarized in Table 3). Earlier studies that purified native LLO directly from *L. monocytogenes *culture supernatants had poor LLO yields (0.022–0.25 mg per litre culture) and utilised laborious multi-step procedures requiring large culture volumes [24,25]. Dealing with such large culture volumes of pathogenic bacteria is potentially hazardous and necessitates special precautions if scaling-up is required. When LLO was constitutively over-expressed and secreted by the non-pathogenic *Listeria innocua*, an improved yield was obtained (1.6 mg LLO per litre culture) [26]. However, purification of LLO from *L. innocua *involved multiple steps involving preliminary concentration by filtration, followed by two sequential chromatography purification steps [26].

**Table 3:** Comparative results of LLO production and purification by different investigators.

**Source of** ** LLO**	**Starter** **Culture** **volume** ** (litre)**	**LLO yield ** **(mg l^-1^) ** **{Total** ** yield (mg)}**	**Specific** **LLO** **haemolytic** **activity (HU** **per mg** **protein) ** ** pH 5.5**	**Expression host**	**Plasmid expression** **system/inducer** ** (If applicable)**	**Reference**
Culture supernatant	27	0.022 {0.6}	10^6 ^(pH 6)	*Listeria monocytogenes*^*a*^	N/A	[24]
Culture supernatant	3	1.6 {4.8}	1.02 × 10^6 ^(pH 5.7)	*Listeria innocua*^*b*^	pERL3-503 (constitutive)	[26]
Culture supernatant	6	0.25 {1.5}	2.6 × 10^5^	LLO-hypersecretor *Listeria monocytogenes*^*a*^	N/A	[25]
Cell lysate	1	4.5 {4.5}	1.25 × 10^6^	*Escherichia coli*^*c*^	pET system/IPTG	[15]
Cell lysate	0.6	3.5 – 8 {2.1 – 4.8}	1.8 × 10^6^	*Escherichia coli*^*d*^	pQE31 system/IPTG	[14]
Cell lysate	0.6	2.5 {1.5}	2.16 × 10^6^	*Escherichia coli*^*e*^	pQE70 system/IPTG	[14]
Cell lysate	0.5	-Single sonication treatment: 4.43 – 5.9 {2.215 – 2.95} -Double sonication approach: 9.3 – 12.9 {4.65 – 6.45}	5 × 10^5 ^– 5 × 10^7^	*Lactococcus lactis *NZ9000^*d*^	NICE system (pNZPnisA:CYTO-LLO)/nisin	This study

(N/A) not applicable; IPTG: Isopropyl β-D-1-thiogalactopyranoside; *a *untagged native full-length LLO; *b *untagged full-length LLO; *c *untagged LLO without signal sequence; *d *N-terminus His-tagged LLO without signal sequence; *e *C-terminus His-tagged LLO without signal sequence

Giammarini and coworkers could inducibly over-express LLO in *E. coli *obtaining a relatively good yield (4.5 mg per litre culture) [15]. However, two sequential purification steps were applied with haemolytic activity assessment on blood agar and SDS-PAGE performed between the first hydroxyapatite purification step and the second ammonium sulphate precipitation step. Further concentration steps were applied before the effluent fractions containing LLO were concentrated by ultrafiltration [15]. Although the LLO yield was good, those multi-step procedures are relatively time-consuming. To the best of our knowledge, Churchill et al were able to obtain the highest yield of haemolytically active LLO [14]. In their study, several expression systems have been attempted in *E. coli *among which the N-terminus His-tagged LLO was the most successful using the pQE31/IPTG (Isopropyl β-D-1-thiogalactopyranoside)-inducible system (Qiagen). This system makes use of the well-characterized strong T5 promoter/*lac *operator transcription-translation system to control LLO expression in *E. coli*. A significant LLO yield (3.5–8 mg per litre culture) was obtained with proven haemolytic activity (1.8 × 10^6 ^HU per mg protein) and stability up to one year upon storage at 4°C [14].

The current *L. lactis*-based LLO production method resembles to some extent the N-terminus His-tagged LLO prepared by Churchill et al [14]. As regards LLO yield, the single sonication treatment gave an appreciably good pure yield (4.43–5.9 mg per litre culture) though the highest value (i.e. 5.9 mg per litre culture) is less than the highest yield obtained by Churchill and coworkers (8 mg per litre culture) [14]. Although the double sonication treatment of the same culture resulted in some minor degradation of LLO (Figure 6), the total yield almost doubled (up to 12.9 mg per litre culture) without using any additional culture. This minor degradation may be tolerable for experimental scale production (but not for large commercial scale production). We also assessed the haemolytic activity of the purified LLO at intervals up to 8 months and found it to be within the same range (5 × 10^5 ^to 5 × 10^7 ^HU per mg total protein).

We consider the most important innovation in the current work is the use of *L. lactis *as an expression host. *L. lactis *is a GRAS, Gram-positive lactic acid bacterium widely used in the food industry. More recently *L. lactis *has demonstrated significant promise as a means of producing heterologous proteins for experimental or commercial applications [8]. Lactic acid bacteria expressing heterologous proteins have been used in the food industry [27], in experimental vaccine antigen delivery [11,28], and for the targeted delivery of therapeutic bioactive proteins (cytokines and trefoil factors) [10,29]. In producing bioactive proteins for human or animal use, lactococci have a significant advantage over *E. coli *in that they do not produce endotoxins. Moreover, *L. lactis *is known to produce very low amounts of native exoproteins and the lactococcal genome is generally about half the size of the *E. coli *genome [9,18,30]. This is an advantage both for *in vitro *heterologous protein production and for the use of *L. lactis *as a vaccine delivery vehicle as there are fewer contaminating proteins when *L. lactis *is used as a heterologous host [18,31].

## Conclusion

The present study offers a convenient method for LLO production in an advantageous host providing a good protein yield, high haemolytic activity and significant stability that compares well with previously published studies. Purified LLO from *L. lactis *may find significant applications in basic research into listerial pathogenesis including the stimulation and development of improved monoclonal antibodies against LLO. This *L. monocytogenes*-specific protein may also have applications in the development of improved food testing protocols. Finally, *L. lactis *production of LLO may have applications in vaccine development.

## Competing interests

The authors declare that they have no competing interests.

## Authors' contributions

MB carried out the molecular genetic and protein studies and drafted the manuscript. BTG participated in the design and coordination of the study. CGMG conceived of the study, and participated in its design and follow-up and helped to draft the manuscript. All authors read and approved the final manuscript.
